# Exploring How Subcutaneous Fat Prevents Pressure Sores: A Study Using Ultrasound and Finite Element Analysis

**DOI:** 10.1055/s-0046-1816058

**Published:** 2026-02-15

**Authors:** Madhubari Vathulya, Praveen AJ, Mayank Mishra, Pankaj Kandwal

**Affiliations:** 1 Department of Burns and Plastic Surgery, All India Institute of Medical Sciences, Rishikesh, Uttarakhand, India; 2 Department of Plastic, Reconstructive and Microvascular Surgery, Rajagiri Hospital, Aluva, Kochi, Kerala, India; 3 Department of Pulmonary Medicine, All India Institute of Medical Sciences, Rishikesh, Uttarakhand, India; 4 Department of Orthopaedics, All India Institute of Medical Sciences, Rishikesh, Uttarakhand, India

**Keywords:** pressure sores prevention, subcutaneous fat thickness, finite element analysis, ultrasound imaging

## Abstract

**Aims:**

This study investigates the protective role of subcutaneous fat in preventing pressure sores, integrating ultrasound and finite element analysis (FEA) to understand the biomechanical mechanisms. It aims to explore the correlation between subcutaneous fat thickness and pressure sore risk as indicated by Braden scale scores.

**Materials and Methods:**

A total of 100 bedridden patients from the Spine Ward and Pulmonary Medicine intensive care unit were enrolled. Subcutaneous fat thickness in the sacral region was measured using ultrasound, and Braden scale scores were recorded. FEA was used to simulate stress and strain distributions in the muscle and fat layers for varying fat thickness. Statistical analysis was performed using analysis of variance, post hoc Tukey test, and Pearson's chi-square test to correlate fat thickness with mechanical parameters.

**Results:**

The results demonstrated a significant negative correlation between subcutaneous fat thickness and pressure sore risk. Patients with lower Braden scale scores (higher risk) had thinner subcutaneous fat. FEA models showed that increased fat thickness reduced maximum total deformation, equivalent stress, and shear stress in muscle and bone tissues.

**Conclusion:**

Subcutaneous fat plays a crucial protective role in preventing pressure sores by reducing mechanical strain on tissues. This study highlights the potential for using subcutaneous fat thickness measurements as a predictive tool for pressure sore risk and suggests the exploration of fat grafting as a preventive strategy.

## Introduction

Pressure sores, also referred to as bedsores, pressure injury, or pressure ulcers, pose significant challenges for individuals with limited mobility. The high cost and complexity of treating pressure sores highlight the crucial need to prevent them in patients at risk, driving extensive research into prevention strategies for this vulnerable population. Studies have identified various risk factors such as age, nutritional status, comorbidities, etc. 1 Recent studies have suggested that subcutaneous fat may play a protective role in the development of pressure sores, highlighting a novel area for preventive intervention. 2

This study extends the existing knowledge base by integrating advanced techniques, specifically ultrasound and finite element analysis (FEA), to comprehensively assess the preventive role of subcutaneous fat in pressure sores. Grounded in patient-centric methodology, the study aims to explore the correlation between local subcutaneous fat thickness and established predictor of bedsores, i.e., the Braden scale. 3 The FEA methodology, rooted in biomechanics and previously employed in studies of tissue deformation, offers insights into stress and strain distributions within the muscle and fat layers during supine positioning. 4 This interdisciplinary approach aims to contribute valuable insights in the development of pressure sores, presenting potential avenues for preventive strategies and improved patient care.

## Materials and Methods

This study assessed the preventive role of subcutaneous fat in pressure sores using a combined approach of ultrasound and FEA. In the clinical observation phase, we correlated subcutaneous fat thickness measured by bedside ultrasonography (USG) in the sacral region with Braden scale scores. The subjects included patients admitted for over 3 days and confined to bed in the spine ward and pulmonary medicine intensive care unit (ICU) of our institute. The inclusion criteria encompassed adults over 18 years with no existing pressure ulcers. The exclusion criteria included patients who had undergone pressure sore surgery, pregnant females, and mentally unstable patients. We limited our sample size to 100, based on the total number of admissions in spine and pulmonary medicine wards over the previous years. Our sample included a heterogenous group of 100 bedridden patients admitted for diverse diagnoses: 19 with spinal cord injury, 39 with pulmonary diseases, and 42 with pulmonary disease and additional comorbidities. The study was started after obtaining approval from the Institutional Ethical Committee.

We collected demographic and clinical information, including age, gender, diagnosis, body weight, height, and length of hospital stay, from all patients. The Braden scale score was assessed for each patient, and subcutaneous fat thickness was measured using bedside USG. The sacral promontory was identified using well-known surface landmarks such as the 10th rib line (corresponding to the L1–L2 intervertebral space) and Tuffier's line (corresponding to the L4–L5 intervertebral space), and intervertebral spaces were counted downward. We analyzed the correlations between local subcutaneous fat thickness in the sacral region and Braden scale scores. The sacral region was selected due to its susceptibility to high pressure when lying supine, making it the most vulnerable area for developing pressure sores.

**Fig. 1:**
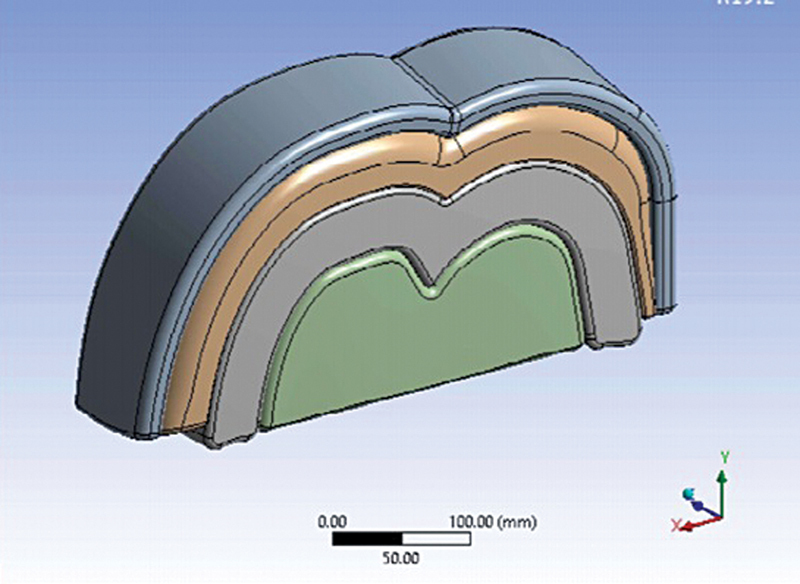
The FEA model. FEA, finite element analysis.

**Fig. 2:**
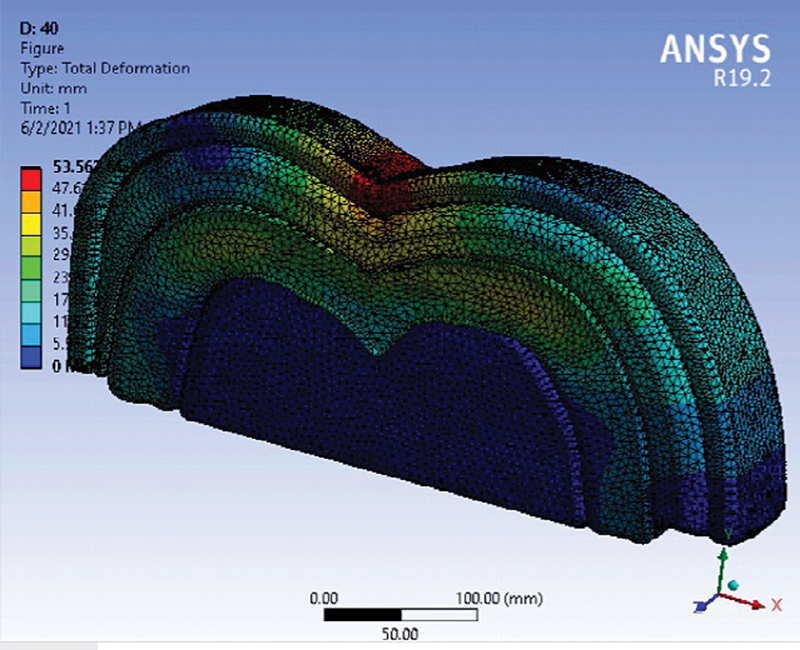
FEA model postdeformation. FEA, finite element analysis.

The FEA methodology involved calculating strain and stress distributions in the muscle and enveloping fat layer in the sacral region while lying supine. We developed a model based on magnetic resonance imaging (MRI) scans of the lower back and gluteal region in both load-bearing supine and nonload-bearing prone positions in a healthy individual after obtaining an informed written consent. An axisymmetric three-dimensional (3D) model of the undeformed gluteal region was constructed ( Fig. 1 ), and subsequent models with varying fat thicknesses (20 mm, 30 mm, 40 mm, 50 mm, 60 mm) were developed using Geomagic Freeform software (version X.X; 3D Systems, Rock Hill, South Carolina, United States). These digitized STL models were transferred to SolidWorks software for FEA. Material parameters for bone, muscle, fat, and skin were defined based on literature values, and Ogden first-order strain energy functions were used for soft tissues, fat, and skin ( Table 1 ). 5 6 Boundary conditions obtained from previous studies were applied, and FEA simulated the forces applied in the supine position 7 ( Fig. 2 ).

**Table 1:** Material parameters for the finite element analysis model

	Bone	Muscle	Fat	Skin
Density	1.4 g/cm ^2^	1.06 kg/L	0.9196 kg/L	1.02 G/cm ^2^
Youngs modulus	16.586 GPa			
Poisson's ratio	0.3			
Mu		1,907 Pa	1,700 Pa	220,000 Pa
Material constant A1		4.6	26	12
Incompressibility parameter		1.05 E − 5	1.18 E − 5	9.12 E − 8

### Validation of the Magnetic Resonance Imaging-Based Three-Dimensional Model of the Buttocks

Lying down induced gross displacement of soft tissues computed by the FEA in the 3D model was compared with the measurements from the MRI taken in supine (lying down) position. The resultant thicknesses of various soft tissue layers postdeformation in the 3D model were remarkably close to that of the measurements of the MRI scan taken in supine position, hence validating our 3D model and the loading and boundary conditions. Our 3D model is the first FEA model for the sacral region validated using actual pre- and postload MRI displacement data, improving over prior models, which were often 2D or used less accurate geometry and boundary conditions.

Parameters such as maximum total deformation, equivalent stress, equivalent strain, shear stress, and shear strain were evaluated for different fat thickness models keeping the interface pressure constant (55 mm Hg). The analysis was repeated for all models of the sacral region with varying fat thicknesses. The developed model was quantitatively validated by comparing measurements postdeformation with MRI images in the supine position.

### Statistical Analysis

Descriptive statistics, including mean ± standard deviation (SD), minimum, and maximum values, were used to portray measurable data. Categorical data were represented as proportions. Analysis of variance (ANOVA) was used to determine the statistically significant differences in subcutaneous fat thickness among the different Braden scale risk groups. Post hoc Tukey Test was then applied to identify which specific groups' means were significantly different from each other. Pearson's chi-square test was used to examine the correlation between FEA parameters and fat thickness. All statistical analyses were conducted using IBM SPSS version 22 software.

## Results

This study included 100 bedridden patients admitted to the Spine Ward and Pulmonary Medicine ICU between July 2019 and July 2021. Among these patients, 66 were male and 34 were female. The illness duration in our cohort ranged from 3 to 60 days. The mean for the entire group was 14.08 days (SD = 14.8, range = 3–70). The mean age was 52.4 years (SD = 15.0, range = 14–85). The average body mass index (BMI) was 22.3 kg/m ^2^ (SD = 3.9, range = 16.3–36.8). Mean hemoglobin was 10.6 g/dL (SD = 2.4, range = 7.2–17.8), and mean serum albumin was 3.12 g/dL (SD = 0.63, range = 1.95–4.8). Mean sacral subcutaneous fat thickness was 2.14 cm (SD = 0.89, range = 1.1–6.3), and mean mid-arm circumference was 28.2 cm (SD = 5.7, range = 16.5–47). Most patients (72.0%) had a Glasgow Coma Scale (GCS) of 15, whereas 28.0% had a GCS < 15. By diagnosis, 39.0% had lung disease (LD), 42.0% had LD with multisystemic involvement (LD + MS), and 19.0% had spinal injury.

Based on Braden scale scores, 34 participants (34.0%) were low risk (score ≥ 15), 44 (44.0%) were moderate risk (score 13–14), and 22 (22.0%) were high risk (score ≤ 12). Table 2 summarizes characteristics by Braden risk category. A one-way ANOVA showed a significant difference in age across groups, *F* (2, 97) = 3.66, *p* = 0.029, η ^2^ = 0.07, with the moderate-risk group being older on average than the low- and high-risk groups. No significant associations were observed between sex and Braden category, χ ^2^ (2, *N* = 100) = 1.63, *p* = 0.443, or between GCS category and Braden category, χ ^2^ (2, *N* = 100) = 4.27, *p* = 0.118. Hemoglobin differed significantly across groups, *F* (2, 97) = 4.09, *p* = 0.020, η ^2^ = 0.08, and serum albumin also varied significantly, *F* (2, 97) = 5.85, *p* = 0.004, η ^2^ = 0.11, with the lowest values in the high-risk group. BMI did not differ significantly across Braden categories, *F* (2, 97) = 0.98, *p* = 0.378, indicating that BMI alone does not discriminate pressure-injury risk as measured by the Braden scale. However, BMI showed a moderate positive correlation with sacral subcutaneous fat thickness, ρ(98) = 0.41, *p* < 0.001, suggesting that higher BMI is associated with greater local fat thickness; nevertheless, direct sacral fat measurement is a better risk discriminator than BMI.

**Table 2:** Distribution of characteristics by Braden scale scores

Characteristics	Low risk (≥15) ( *n* = 34)	Moderate risk (13–14) ( *n* = 44)	High risk (≤12) ( *n* = 22)	*p* -Value
Age, mean (SD)	47.6 (16.5)	56.6 (12.8)	51.2 (15.1)	0.029
Sex, *n* (%)	0.443			
Male	21 (61.8)	32 (72.7)	13 (59.1)	0.443
Female	13 (38.2)	12 (27.3)	9 (40.9)	
GCS, *n* (%)	0.118			
15	26 (76.5)	34 (77.3)	12 (54.6)	0.118
< 15	8 (23.5)	10 (22.7)	10 (45.5)	
BMI, mean (SD)	23.0 (3.8)	22.1 (3.7)	21.7 (4.3)	0.378
Hemoglobin, mean (SD)	11.1 (2.1)	10.7 (2.7)	9.4 (1.6)	0.020
Albumin, mean (SD)	3.30 (0.66)	3.16 (0.62)	2.75 (0.48)	0.004
Diagnosis, *n* (%)	0.008 ^a^			
LD	13 (38.2)	23 (52.3)	3 (13.6)	0.008
LD + MS	15 (44.1)	17 (38.6)	10 (45.5)	
SI	6 (17.6)	4 (9.1)	9 (40.9)	

Abbreviations: BMI, body mass index; GCS, Glasgow Coma Scale; LD, lung disease; MS, multisystemic involvement; SD, standard deviation; SI, spinal injury.

**Fig. 3:**
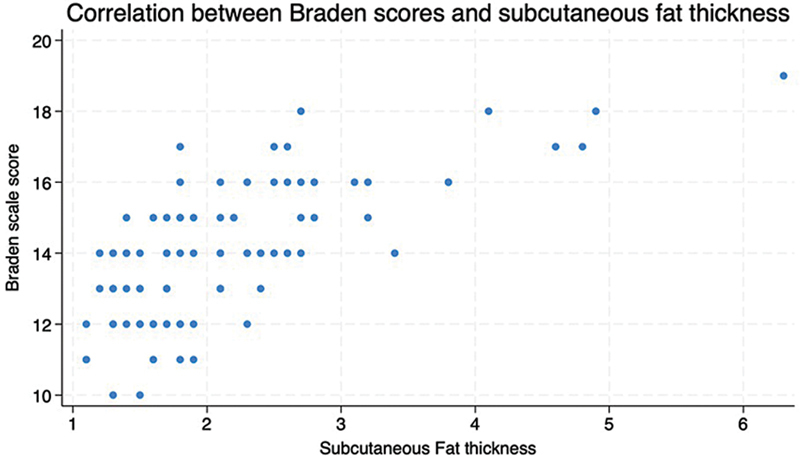
Correlation between Braden scores and subcutaneous fat thickness.

Table 3 presents subcutaneous fat thickness by Braden category. A one-way ANOVA revealed a significant effect, *F* (2, 97) = 23.05, *p* < 0.001, η ^2^ = 0.32 (large effect). Post hoc Scheffé tests showed that the low-risk group (M = 2.81 cm, SD = 1.10) had greater fat thickness than the moderate-risk group (M = 1.90 cm, SD = 0.51, *p* < 0.001) and the high-risk group (M = 1.56 cm, SD = 0.30, *p* < 0.001); the moderate- and high-risk groups did not differ ( *p* = 0.210). A Spearman rank-order correlation further showed a strong positive association between sacral subcutaneous fat thickness and Braden scores, ρ(98) = 0.66, *p* < 0.001, indicating that greater sacral fat thickness is associated with higher (more favorable) Braden scores ( Fig. 3 ).

**Table 3:** Comparison of subcutaneous fat thickness (cm) across Braden risk groups

Braden risk group	*n*	Mean ± SD (cm)	ANOVA *F* (2,97)	Post hoc Scheffé ( *p* -Value)
Mild risk (≥15)	34	2.81 ± 1.10	*F* = 23.05, *p* < 0.001	vs. moderate: *p* < 0.001 ^a^
Moderate risk (13–14)	44	1.90 ± 0.51		vs. high: 0.210 (NS)
High risk (≤12)	22	1.56 ± 0.30		vs. mild: *p* < 0.001 ^a^

Abbreviations: ANOVA, analysis of variance; NS, not significant; SD, standard deviation.

Notes: The *F-* value (ANOVA) pertains to the overall variance among the groups. The Post hoc Scheffé Test values show the significance levels for pairwise comparisons between the groups.

**Fig. 4:**
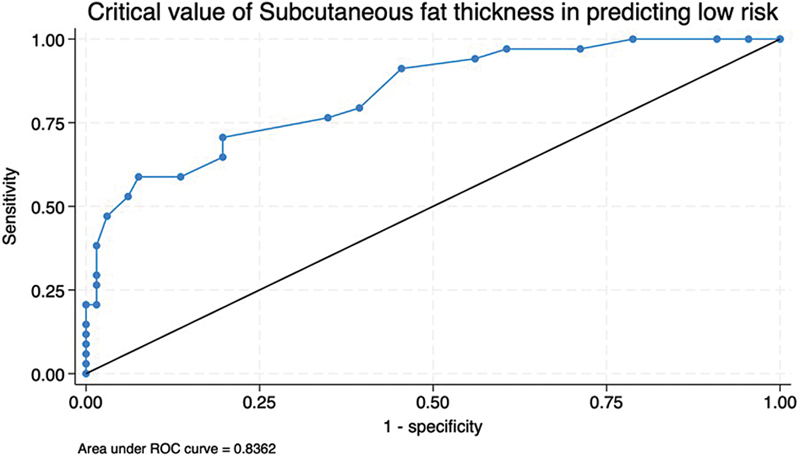
Receiver operating characteristic curve for subcutaneous fat thickness in predicting low-risk patients.

A receiver operating characteristic (ROC) analysis ( Fig. 4 ) evaluated the ability of sacral subcutaneous fat thickness to identify low-risk patients (Braden ≥ 15) versus moderate-to-high risk (≤14). Discrimination was good: AUC = 0.84, 95% CI [0.75, 0.92], *p* < 0.001. A threshold of approximately 2.2 cm provided the most balanced classification, correctly classifying approximately 77% of patients. Clinically, patients with thickness below approximately 2.2 cm appear substantially more vulnerable and may warrant intensified preventive care, whereas those above approximately 2.2 cm are more likely to be protected. Higher thresholds (>2.5 cm) reduce false positives but miss some vulnerable patients; lower thresholds (<1.5 cm) capture nearly all at-risk cases but are less accurate overall. Thus, approximately 2.2 cm is a practical, clinically useful threshold rather than an absolute “critical” value.

### Results of Finite Element Analysis

#### Maximum Total Deformation

**Fig. 5:**
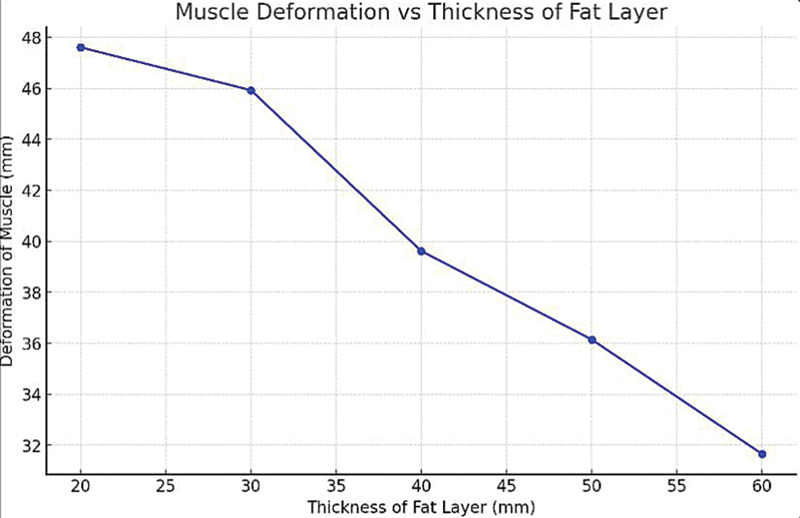
Muscle deformation versus thickness of fat layer.

Maximum total deformation refers to the greatest amount of distortion or displacement experienced by a material or structure under load. The study observed a declining trend in the maximum deformation values of muscle tissue as fat thickness increased. Specifically, the muscle layer in the model with a fat thickness of 60 mm exhibited the lowest maximum total deformation at 31.658 mm, whereas the model with a fat thickness of 20 mm recorded the highest deformation at 47.607 mm. Additionally, a significant negative correlation ( *p* = 0.001) was found between fat thickness and maximum total deformation in muscle layer ( Fig. 5 ).

#### Equivalent Stress

Equivalent stress represents the combined effect of various stresses (such as tension, compression, and shear) acting simultaneously on a material. A noteworthy negative correlation was found between fat thickness and equivalent stress in both muscle and skin layers, with *p* -values of 0.168 and 0.223, respectively. The bone layer showed a statistically significant negative correlation with fat thickness ( *p* = 0.04), indicating a strong relationship between fat thickness and equivalent stress in bone.

**Fig. 6:**
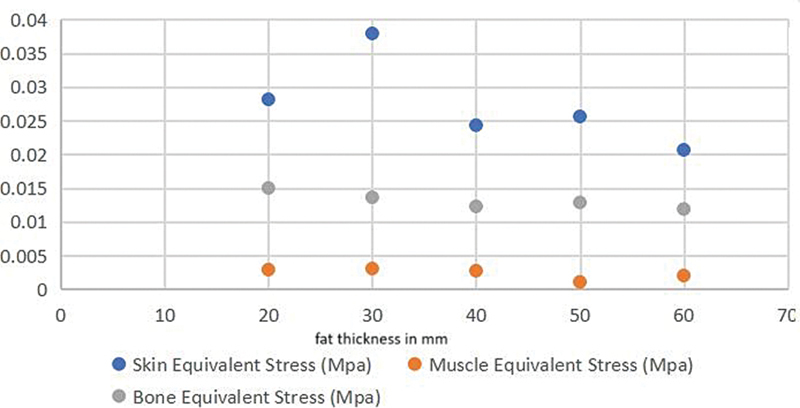
Equivalent stress in various layers of tissue at varying thickness of fat layer.

These findings suggest that as fat thickness increases, the equivalent stress in muscle and skin tissues decreases, implying that thicker fat layers may provide a cushioning effect that reduces stress on these tissues. Additionally, the significant negative correlation between fat thickness and equivalent stress in bone indicates that higher fat thickness is associated with lower stress levels in bone tissue ( Fig. 6 ).

#### Equivalent Strain

**Fig. 7:**
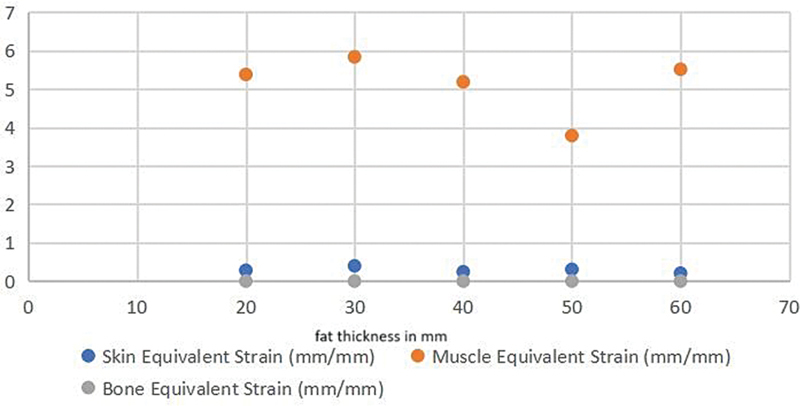
Equivalent strain in various layers of tissue with varying thickness of fat layer.

When a material is subjected to loading, it may experience various types of strain simultaneously, such as elongation, compression, shearing, and bending. Equivalent strain provides a unified measure that accounts for the combined effect of these different strain components. Equivalent strain varied in muscle and skin layers with fat thickness. No significant correlation existed between fat thickness and equivalent strain in skin or muscle ( *p* = 0.357, 0.558). Similarly, bone showed no significant correlation ( *p* = 0.149; Fig. 7 ).

#### Shear Stress

**Fig. 8:**
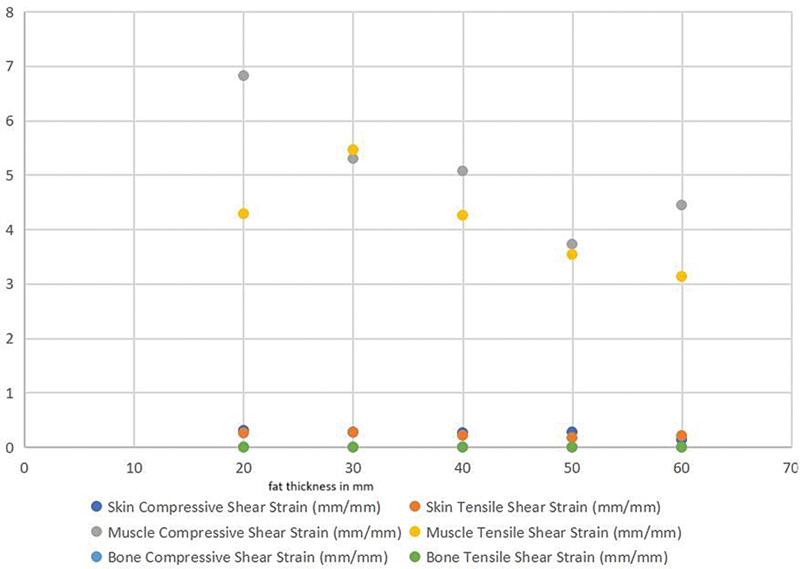
Shear strain in various layers of tissue with varying thickness of fat layer.

Shear stress is a type of stress that occurs when adjacent layers of a material or structure slide past each other in opposite directions. Increasing fat thickness led to a decline in compressive shear stress in the muscle layer. The highest stress (0.00138 MPa) occurred at 20 mm fat thickness, and the lowest (0.0009 MPa) at 60 mm. While skin compressive shear stress showed a good negative correlation, it was not statistically significant ( *p* = 0.067). However, skin tensile shear stress exhibited a significant negative correlation ( *p* = 0.013). Muscle compressive and tensile shear stresses displayed excellent negative correlations but were not statistically significant ( *p* = 0.057 and 0.142, respectively). Bone compressive and tensile shear stresses showed significant negative correlations ( *p* = 0.015 and 0.027, respectively; Fig. 8 ).

#### Shear Strain

**Fig. 9:**
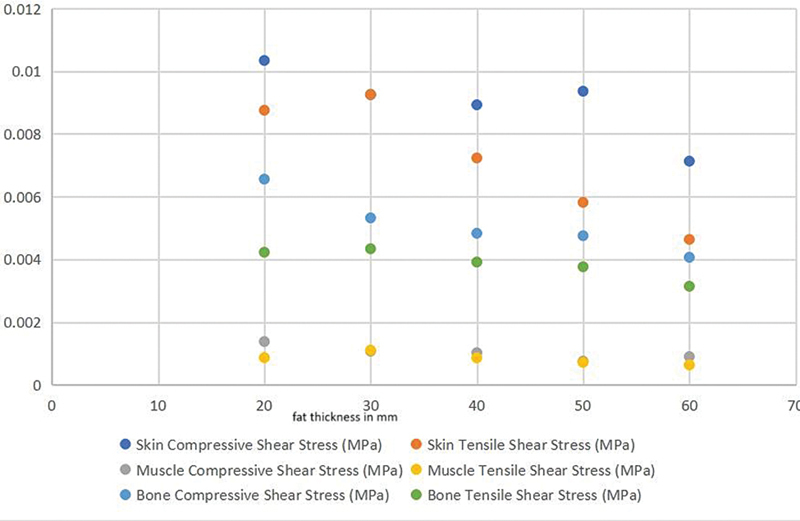
Shear stress in different layers of tissue with varying thickness of fat layer.

Shear strain is a measure of deformation that occurs when adjacent layers of a material or structure slide past each other in opposite directions. Fat thickness correlates negatively with skin, muscle, and bone shear strains, though not significantly for skin and muscle ( *p* = 0.102, 0.13, 0.057, 0.142), but considerably for bone ( *p* = 0.014, 0.027). This suggests that as fat thickness increases, there is a negative correlation with shear strains in the skin, muscle, and bone layers ( Fig. 9 ).

## Discussion

The findings from this investigation underscore the critical role of subcutaneous fat in mitigating the risk of pressure sores. By employing both ultrasound and FEA, we have provided a nuanced understanding of how varying fat thicknesses influence the mechanical stress and strain distributions within the sacral region.

Our study supports earlier findings that subcutaneous fat helps protect against pressure sores. 2 The ultrasound measurements revealed a significant correlation between increased subcutaneous fat thickness and higher Braden scale scores, indicating a lower risk of pressure sore development. This suggests that subcutaneous fat may have a cushioning effect, reducing stress on the underlying muscle. Since subcutaneous fat thickness can be easily measured at the bedside using USG, this method can be utilized in all clinical settings as an additional tool to predict the risk of pressure sore development in at-risk immobile patients. Additionally, considering this perspective, we might explore whether fat grafting in this region for at-risk patients could prevent pressure sore formation. This question warrants further investigation. Previnaire et al. reported reduced recurrence of pressure sores in the ischial region in already operated cases by identifying at-risk patients using Braden scale scores and skinfold thickness assessments, followed by performing lipofilling in such patients. 8 Marangi et al. used USG to assess patients at risk for pressure sores, they also did fat grafting in patients with grade 1 pressure sores thus halting their progression. 9

The FEA models provided a detailed biomechanical perspective by simulating the stress and strain distributions across different fat thickness scenarios. Our findings showed that thicker subcutaneous fat layers resulted in lower maximum total deformation, equivalent stress, and equivalent strain in the muscle and fat tissues. This supports the hypothesis that subcutaneous fat buffers mechanical forces, thereby preventing tissue deformation and subsequent ulceration. 10 Furthermore, the detailed analysis of stress and strain distributions offers critical insights into how mechanical forces are transmitted through different tissue layers. This information is invaluable for designing better supportive surfaces, such as mattresses and cushions, that can enhance patient comfort and reduce the incidence of pressure sores. 11 12 The adaptability and precision of our FEA model make it a valuable tool for further research. Future studies can incorporate patient-specific data, such as varying tissue properties and individual body morphologies, to enhance the predictive accuracy of the model. This personalization can lead to better-tailored preventive measures for individuals at high risk of developing pressure sores. Moreover, the integration of FEA with real-time monitoring technologies could lead to the development of dynamic, adaptive support surfaces that respond to the changing needs of patients. For example, pressure mapping sensors combined with FEA simulations can provide continuous feedback, allowing for immediate adjustments to be made to patient positioning or support surface characteristics.

### Clinical Implications

USG is a cost-effective, noninvasive bedside tool that provides objective data for assessing pressure sore risk. While the Braden scale is widely used and clinically valuable, its subjectivity can limit predictive accuracy. Integrating sacral fat thickness measurements via USG may improve risk stratification by adding quantifiable parameters. Clinically, this supports incorporating USG into routine evaluations for at-risk patients, enabling personalized preventive strategies—such as targeted nutritional support to maintain adequate subcutaneous fat levels. 13 Its accessibility and ease of use also make USG ideal for regular monitoring, allowing timely adjustments to care plans and potentially reducing the incidence of pressure ulcers, thereby improving patient outcomes. 14

Our study has added to the existing evidence that subcutaneous fat has a protective role in preventing pressure sores. Our group demonstrated using three-dimensional scanning techniques that fasciocutaneous flaps, which predominantly contain subcutaneous fat, were better suited for covering defects caused by sacral pressure sores. 15 A systematic review by our group has shown a significant difference in recurrence rates between pressure sores covered by fasciocutaneous flaps and those covered by muscle flaps. 16 Studies have shown that fat grafting can be used to treat both earlier and later stages of pressure sores. 8 9 All this evidence supports the development of clinical guidelines that incorporate subcutaneous fat assessment by USG as a standard practice in pressure sore risk management. Additionally, it suggests further research into fat grafting for at-risk patients to prevent the formation of pressure sores. 17 Future studies should also investigate the interplay between subcutaneous fat and other risk factors, such as comorbidities and nutritional deficiencies, to develop a more comprehensive understanding of pressure sore etiology.

## Limitations

Despite the promising results, our study has several limitations. The observational design and the specific patient population may limit the generalizability of the findings. Clinical outcomes were not tested due to the study's cross-sectional/observational design. The study does not account for individualized differences in lumbosacral fat distribution or spinal curvature; the FEA model is based on a single healthy volunteer's MRI scan. Future research should aim to replicate these findings in diverse cohorts and explore longitudinal outcomes. Additionally, the development of more sophisticated FEA models incorporating dynamic loading conditions could provide deeper insights into the preventive mechanisms of subcutaneous fat. 18 In this study, the MRI-based model tested the changes in muscle by varying only subcutaneous fat thickness, whereas muscle parameters were kept constant. Future models should incorporate variations in muscle atrophy, patient-specific anatomy, and tissue properties like fat infiltration.

## Conclusion

This study demonstrates that sacral subcutaneous fat plays a significant biomechanical and clinical protective role against pressure sore development, as evidenced through both bedside USG and validated FEA. Greater fat thickness strongly correlated with higher Braden scores and lower risk categories, and ROC analysis identified a critical sacral fat thickness of approximately 2.2 cm as a practical threshold that reliably distinguishes low-risk patients from those at moderate–high risk. FEA further confirmed that increasing fat thickness markedly reduces maximum total deformation, equivalent stress, and shear stress and strain in muscle, skin, and bone—highlighting the cushioning effect of fat in redistributing mechanical loads during prolonged supine positioning. These findings support incorporating sacral fat-thickness measurement via USG—an objective, accessible, bedside tool—into routine risk assessment to complement the subjective Braden scale and enable more accurate, individualized prevention strategies. The results also reinforce emerging evidence that fat augmentation (fat grafting/lipofilling) may benefit patients with insufficient sacral padding, given the clear biomechanical advantage conferred by greater subcutaneous fat. Overall, a sacral fat thickness below approximately 2.2 cm identifies a vulnerable population requiring intensified preventive measures, while thicknesses above this level offer increasing protection, making USG fat measurement a valuable addition to pressure-sore risk prediction and future interventional research.
